# A ricin-based peptide BRIP from *Hordeum vulgare* inhibits M^pro^ of SARS-CoV-2

**DOI:** 10.1038/s41598-022-15977-y

**Published:** 2022-07-27

**Authors:** Prakriti Kashyap, Vijay Kumar Bhardwaj, Mahima Chauhan, Varun Chauhan, Asheesh Kumar, Rituraj Purohit, Arun Kumar, Sanjay Kumar

**Affiliations:** 1 Biotechnology Division, CSIR-Institute of Himalayan Bioresource Technology, Palampur, Himachal Pradesh 176061 India; 2 Academy of Scientific and Innovative Research, Ghaziabad, 201002 Uttar Pradesh India; 3 Structural Bioinformatics Lab, CSIR-Institute of Himalayan Bioresource Technology, Palampur, Himachal Pradesh 176061 India; 4 Covid-19 Testing Facility, Dietetics & Nutrition Technology Division, Council of Scientific and Industrial Research-Institute of Himalayan Bioresource Technology (CSIR-IHBT), Palampur, H.P India 176061

**Keywords:** Predictive medicine, Molecular medicine

## Abstract

COVID-19 pandemic caused by SARS-CoV-2 led to the research aiming to find the inhibitors of this virus. Towards this world problem, an attempt was made to identify SARS-CoV-2 main protease (M^pro^) inhibitory peptides from ricin domains. The ricin-based peptide from barley (BRIP) was able to inhibit M^pro^ in vitro with an IC_50_ of 0.52 nM. Its low and no cytotoxicity upto 50 µM suggested its therapeutic potential against SARS-CoV-2. The most favorable binding site on M^pro^ was identified by molecular docking and steered molecular dynamics (MD) simulations. The M^pro^-BRIP interactions were further investigated by evaluating the trajectories for microsecond timescale MD simulations. The structural parameters of M^pro^-BRIP complex were stable, and the presence of oppositely charged surfaces on the binding interface of BRIP and M^pro^ complex further contributed to the overall stability of the protein-peptide complex. Among the components of thermodynamic binding free energy, Van der Waals and electrostatic contributions were most favorable for complex formation. Our findings provide novel insight into the area of inhibitor development against COVID-19.

## Introduction

With the emergence of SARS-CoV-2 (Severe Acute Respiratory Syndrome) as a highly contagious virus causing coronavirus disease (COVID-19), the world is facing a pandemic. For the past two decades, humankind has witnessed major outbreaks of fatal human pneumonia caused by three human coronaviruses, including SARS, MERS, and SARS-CoV-2^1^. However, SARS-CoV-2 has caused maximum damage to the human race. Since its first discovery in December 2019 in China, there has been no stopping of the disease. It breached all boundaries spreading to almost all continents, with more than 537.59 million confirmed cases and 6.32 million deaths worldwide as of 20^th^ June 2022 (https://covid19.who.int/). The SARS-CoV-2 is an enveloped, positive-sense, -stranded RNA virus encoded by a genome of about 30,000 nucleotides^2^. Upon entering the host cell, SARS-CoV-2 uncoats and starts transcription and translation^3^. Its genome encodes two polyproteins, namely, ppa1 and pp1ab, which are proteolyzed into non-structural proteins (nsps) by 3C-like protease (3Cl^pro^) also known as main protease (M^pro^) and papain-like protease (Pl^pro^)^4^. These functional nsps, including RNA-dependent RNA polymerase (RdRp) and helicases, are further responsible for genome replication and protein synthesis^5^. M^pro^, a cysteine protease, mediates the maturation and cleavage of polyproteins^6^. Therefore, its inhibition can affect the proliferation of the virus by blocking viral RNA replication and transcription^5^. Because of its potential to inhibit the proliferation of coronavirus and the absence of its homologs in the human genome, it has been considered a major hotspot for drug development by several research groups^5^.

Peptides and peptide-derived inhibitors are other attractive alternatives to drug molecules due to their target specificity, effectiveness, safe nature, and ease of synthesis^7^. The inhibitors that mimic natural peptides known as peptidomimetics were reported to bind M^pro^ in SARS-CoV-2, leading the warhead group to catalyze the formation of cysteine-participating covalent bonds^8,9^. Antiviral peptides can interact with their target proteins, induce conformational changes, and/or modulate their function to inhibit viral replication. One example includes ribosomal inactivating proteins (RIPs) that have shown efficacy against several viral diseases in plants and animals. RIPs belong to a toxin family of proteins with ricin domains. Two types of RIPs have been reported: type I RIP with a single polypeptide chain (Chain A) that has ribosomal inactivating property and type II RIP with additional polypeptide (Chain B) containing lectin domain. The presence of chain B was considered to be responsible for cell toxicity. A single-chain RIP from *Phytolacca americana* was identified as an antiviral protein (PAP)^10–12^. Similarly, trichosanthin and momorcharin are known inhibitory proteins against HIV replication^13,14^. Moreover, two other members, luffin and saporin were proved as HIV inhibitory proteins credited to their HIV-1 integrase inhibition^14^. A short peptide (33AA) of GAP31 (gelonium anti-HIV protein of 31 kDa) is a peptide with ribosomal inactivation potential that elicited anti-HIV effects^15^. Recently, RIP family members like saporin and RTAM-PAP1 were discussed to have therapeutic potential against COVID-19^16,17^.

Despite their antiviral properties, the therapeutic potential of the RIP family is not well translated. This motivated us to evaluate the efficacy of ricin-based peptides against SARS-CoV-2. Computational drug biologists widely use molecular docking and dynamics to screen the molecules with better affinity and promise inhibitory activities towards the target protein. In the present study, the inhibitory peptides from known antiviral RIPs along with barley RIP (BRIP) were designed and screened for their physicochemical properties. BRIP with the lowest allergenicity was evaluated for its inhibitory potential against M^pro^ of SARS-CoV-2. The molecular docking and dynamics approach was further adopted to investigate the potential binding pattern between barley RIP and M^pro^. Docking and steered molecular dynamics (MD) simulations were used to identify the peptide's most potent binding site on M^pro^. Further, conventional MD simulations were utilized for analyzing protein-peptide interactions and observed structural perturbations in M^pro^ due to the binding of the peptide. Moreover, the post-processing thermodynamic binding free energy between M^pro^ and BRIP was also calculated by the widely acceptable Molecular Mechanics Poisson-Boltzmann Surface Area (MMPBSA) approach.

## Results and discussion

Ricin has contributed as a therapeutic plant-based toxin for biomedical applications. Here, the potential of ricin-based peptides against the causal agent of current pandemic SARS-CoV-2 was evaluated.

### Ricin peptides and their physicochemical properties

**Figure 1:**
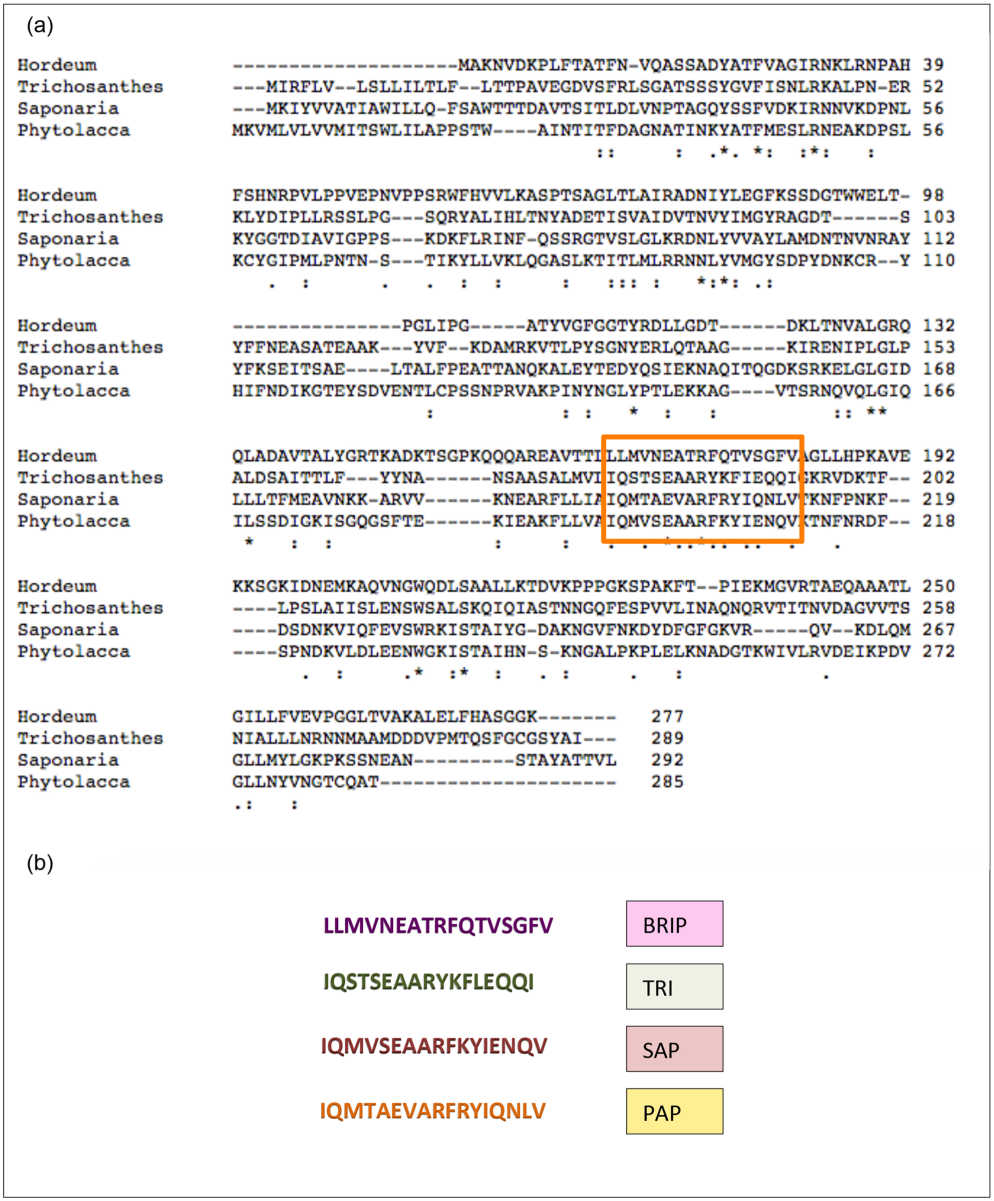
The multiple sequence alignment of type-I RIPs; an antifungal RIP1 from barley; *Hordeum vulgare* (accession number KAE8814914.1), trichosanthin from *Trichosanthes kirilowii* (accession number AAA34207.1), saporin from *Saponaria officinalis* (accession number CAA41948.1), PAP-S1 from *Phytolacca americana* (accession number KT630652.1.) with conserved ricin/shiga toxin domain (predicted by ExPASY PROSITE) boxed (**a**). (**b**) The sequences of ricin- peptides retrieved from barley RIP (BRIP), trichosanthin (TRI), saporin (SAP) and PAP-S1 (PAP).

Four ricin peptides, PAP, SAP, TRI, and BRIP, were designed on the basis of the ricin domain of PAP-S1 (*Phytolacca americana*), saporin (*Saponaria officinalis*), trichosanthin (*Trichosanthes kirilowii*) and an antifungal RIP1 (*Hordeum vulgare*) (Fig. 1). Based on allergenicity results predicted by AlgPred, the peptide BRIP was observed to be non-allergen with a very low allergenicity score of − 0.57, and the rest of the peptides were predicted as allergens with scores of 0.17, 0.037 and − 0.17 for PAP, SAP, and TRI, respectively (Table S1). The allergenicity of known ricin proteins has constrained their therapeutic role. The extremely low allergenicity score of BRIP indicates it to be a non-toxic peptide and might improve its candidature for therapeutic studies. To validate BRIP as a potential SARS-CoV-2 inhibitor, we studied its effect on the inhibition of M^pro^, a protein crucial for viral replication.

### BRIP inhibits M^pro^

**Figure 2:**
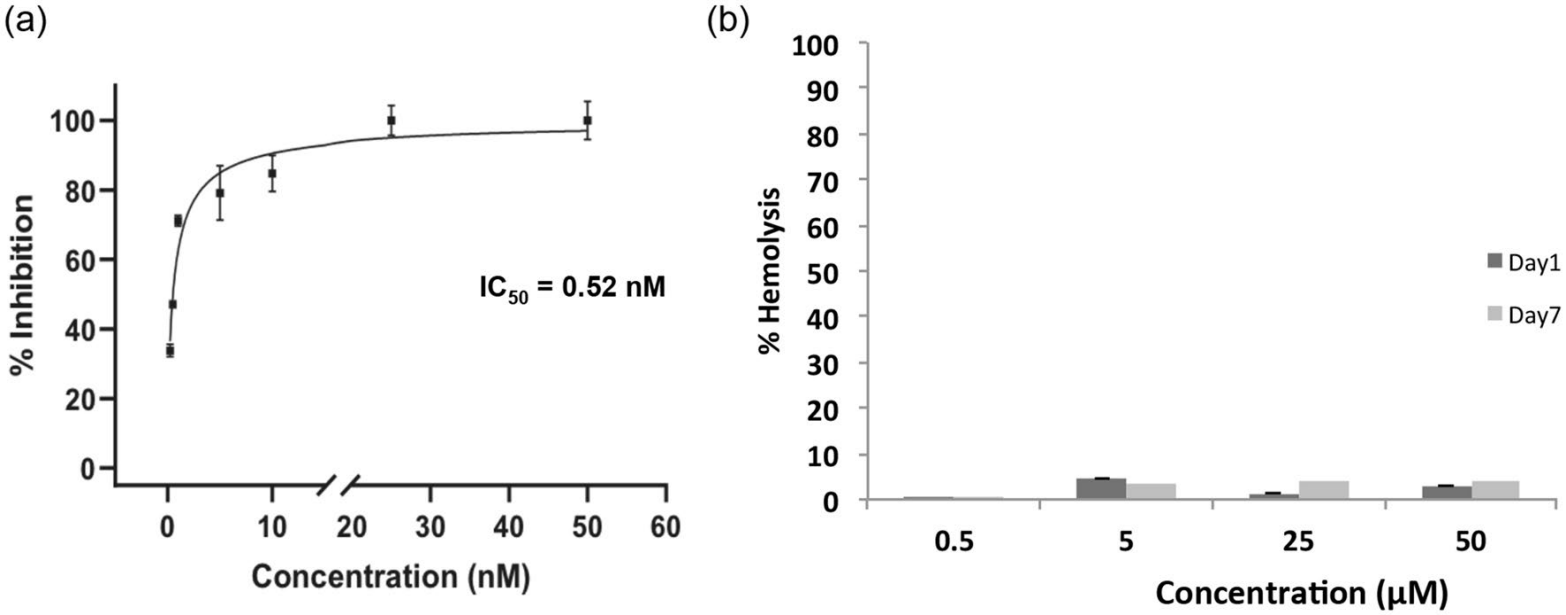
Effect of BRIP on inhibition of M^pro^ activity and determination of cytotoxicity. (**a**) The IC_50_ value (0.52 nM) of BRIP was determined by studying the inhibition of M^pro^ at different test concentrations, as described in the Methods section. (**b**) Percent hemolysis of rat erythrocytes by BRIP at different concentrations. Data represent mean ± SE, n = 3 independent replicates.

The inhibition assay of M^pro^ was performed by incubating it with 0.25–50 nM BRIP for 30 min. BRIP (10–50 nM) as an inhibitor resulted in complete inhibition of M^pro^. The IC_50_ of the peptide against M^pro^ was calculated to be 0.52 nM, a significantly lower concentration (Fig. 2a). This indicated that BRIP is a potential SARS-CoV-2 inhibiting peptide. Earlier, Huang et al.^18^ reported the potential of small peptides in disrupting the interaction between ACE2 and SARS CoV-2 spike protein. Furthermore, peptide and peptide-based inhibitors were screened for their efficacy against SARS-CoV-2, targeting the spike protein of the virus^19^. These studies were oriented toward restricting the viral entry into the cells. However, with rapid mutations reported in the SARS-CoV-2 virus, the peptide inhibitors of M^pro^ are being investigated. A cyclic peptide inhibitor that mimics the conformation of a substrate at a C-terminal autolytic cleavage site of M^pro^ was investigated, and a modest antiviral activity with IC_50_ of 160 µM was found^20^. With an IC_50_ of 0.52 nM, BRIP is a potential M^pro^ inhibitory peptide.

### BRIP is not hemolytic

The therapeutic potential of BRIP will not be useful if it is cytotoxic to cells. Therefore, the cytotoxicity studies on erythrocytes as a model were performed. The mammalian erythrocytes are used as a model for the evaluation of cytotoxicity in xenobiotic and pharmacological studies^21^. The RBCs being convenient to find and handle, are becoming a popular choice for cytotoxicity studies. The damage to RBCs causes their lysis and releases hemoglobin, which can be measured in hemolysis. Hemoglobin can be quantified and is directly proportional to the extent of damage caused to the cells. The potential of erythrocytes in biomedical, pharmaceutical, and toxicology science was shown by Podsiedlik et al.^21^. In the present study, the rat erythrocytes were used to analyze the toxicity of BRIP in erythrocytes. BRIP in concentrations ranging from 1 to 100 µg/ml (500 nM–50 µM) was used for storing RBCs upto seven days. The hemolysis assay revealed that BRIP is not hemolytic at 500 nM and slightly hemolytic at 50 µM (Fig. 2b). As IC_50_ of BRIP against M^pro^ is 0.52 nM, a substantial difference in M^pro^ inhibiting concentration and cytotoxic concentration makes BRIP a potential therapeutic solution to COVID-19. Therefore, BRIP qualifies as a potential inhibitor. To further evaluate the mechanistic anti-M^pro^ activity of BRIP, in silico analysis was performed.

### Modeling of BRIP and detection of M^pro^ binding site

**Figure 3:**
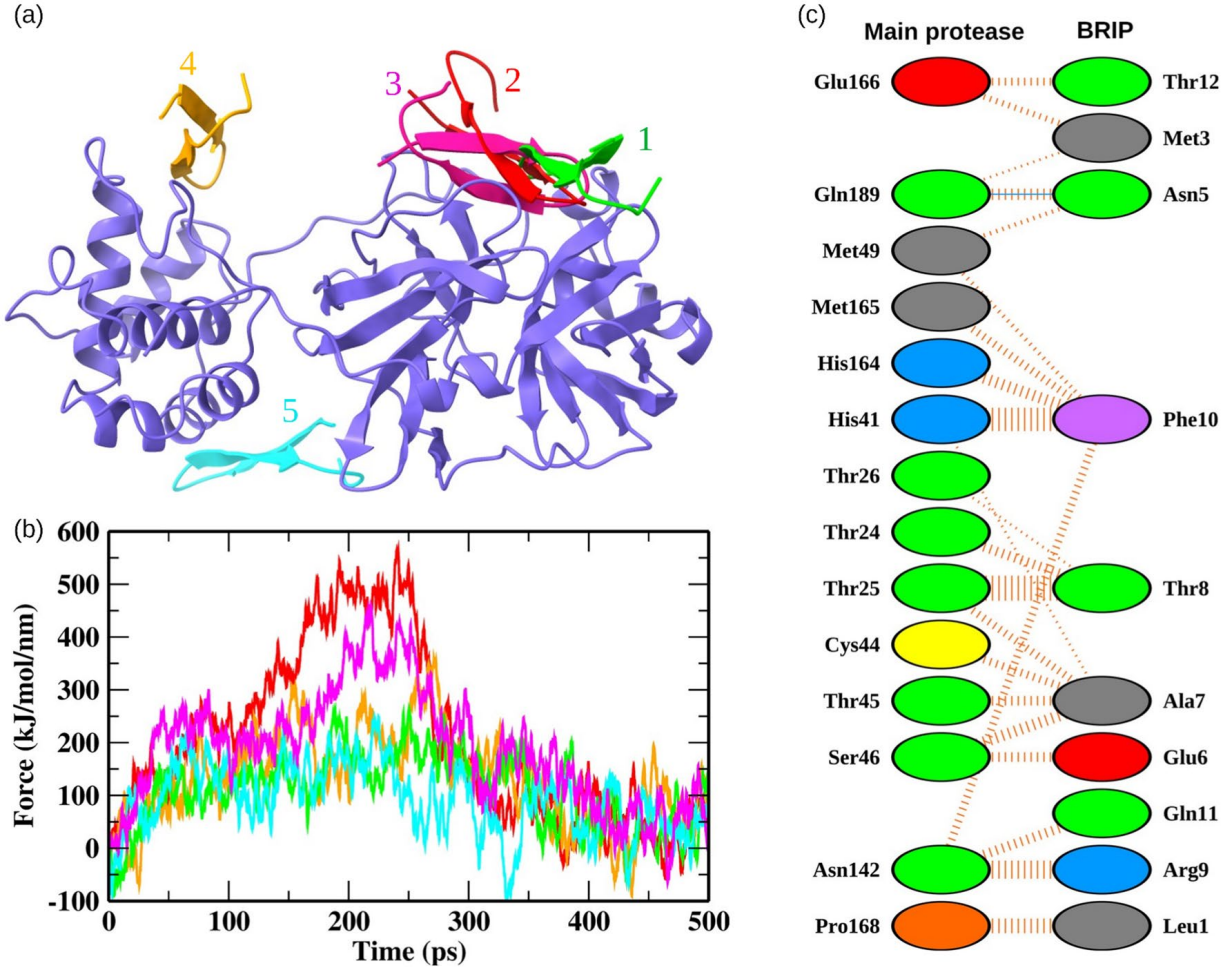
Detection of the protein-peptide binding site. (**a**) BRIP docked on the top five predicted binding sites on M^pro^. (**b**) The pull force profiles of BRIP attached to the binding pockets of M^pro^. The pull force trajectories are colored according to the binding poses shown in panel a. (**c**) The M^pro^-BRIP interactions at the most favorable binding site. The solid blue color lines represent hydrogen bonds, while the striped lines denote non-bonded interactions. The residue color coding scheme: positive (blue), negative (red), neutral (green), aliphatic (gray), aromatic (purple), proline and glycine (orange).

The 3D structure of BRIP was predicted by the online server I-TASSER and the best model based on confidence score, TM score, and RMSD values was selected. The model was further refined by Galaxy refine server, and the reliability of the finalized model was checked by Ramachandran plots (Fig. S1). A prerequisite for the development of potential drug candidates is detecting druggable and functionally significant binding sites^22^. Hence, we detected the potential binding pockets on the surface of M^pro^ of SARS-CoV-2 by exploring the receptor cavities through the Discovery studio package^23^. The top five pockets were selected, and the BRIP peptide was docked on each binding site (Fig. 3a). The residues belonging to these five binding sites are shown in Table S2. The binding site with the highest affinity was determined by employing steered MD simulations. An external pulling by a virtual damped harmonic spring was applied to the BRIP peptide bound to the five predicted binding sites. The maximum external force (F_max_) applied to completely unbind BRIP was used to rank the binding affinity of BRIP peptide for each binding pocket (Fig. 3b, Movies S1–S5). The relationship between the pull force profiles and the unbinding distance for all the M^pro^-BRIP complexes is shown in Fig. S2. The binding pocket 2 experienced the highest F_max_ of 563.52 kJ/mol/nm at ~ 240.8 ps, while the F_max_ experienced by pocket 3 (close to pocket 2) was 450.31 kJ/mol/nm at ~ 219.5 ps. These results showed that BRIP was bound to pocket 2 with the highest binding affinity and also validated the docking scores. The binding pocket 2 shared residues with the binding site targeted by small molecules to inhibit the M^pro^ of SARS-CoV-2^4,24,25^. Further, we explored the binding pattern of BRIP with the predicted binding pocket by molecular docking and also analyzed the dynamics of protein-peptide binding by conventional MD simulations.

### Analysis of M^pro^-BRIP interactions

The molecular interactions between BRIP and M^pro^ at the predicted binding pocket were analyzed, as shown in Fig. 3c. The number of interface residues of M^pro^ and BRIP was 14 and 10, respectively. The average interface area between both the chains was 621.5 Å^2^. BRIP showed a total of 113 non-bonded interactions and one hydrogen bond with Gln189 of M^pro^. The width of the striped lines is proportional to the number of atomic contacts for non-bonded interactions. BRIP showed a non-bonded contact with His41. The residue His41, along with Cys145, forms the catalytic dyad between domain I and domain II of M^pro^^4^. BRIP also exhibited non-bonded contacts with residues from the S1 subsite (Asn142, Glu166, His164, and Met165) of the conventional binding pocket of M^pro^. Targeting the S1 subsite was shown to improve the binding affinity of molecules for the binding pocket of M^pro^^26^. The BRIP peptide also interacted with the conserved residues Gln166, Asn142, His141, and Asn189^4,27^, indicating it to be a promising candidate for inhibition of M^pro^. Further, we employed conventional MD simulations to validate the binding pose and visualize the dynamics involved in protein-peptide binding.

### M^pro^ and BRIP formed a stable protein-peptide complex

The protein-peptide complex was subjected to long-term (1 µs) explicit solvent MD simulations. We calculated the structural properties, including root mean square deviations (RMSD), root mean square fluctuations (RMSF) of backbone Cα atoms, radius of gyration (Rg), and solvent accessible surface area (SASA) of the M^pro^-BRIP complex to analyze the stability of simulations. RMSD is an indicator of the global fluctuations/structural stability of the protein–ligand/peptide complexes subjected to MD simulations^28,29^. We observed an initial increase in RMSD values till ~ 200 ns, followed by a slight dip in the trajectory, and eventually, the RMSD values stabilized after ~ 800 ns (Fig. S3a). The average RMSD over the entire simulation run for the M^pro^-BRIP complex was 0.38 nm. Fluctuation changes between 0 and 0.5 nm are perfectly acceptable for small proteins^28,30^. The simulation's minor fluctuations and well-equilibrated RMSD trajectory suggested a stable protein-peptide complex. The low RMSD values also validate the robustness of the docking protocol followed for the generation of protein-peptide complexes. We extracted the root mean square fluctuation (RMSF) values for the M^pro^-BRIP complex to present fluctuations at a residual level. The residues involved in protein-peptide interactions showed minimal fluctuations during the simulation (Fig. S3b). The residues showing fluctuations over 0.25 nm are considered to belong to flexible regions. The low RMSF values for interacting residues suggested stable interactions between protein and peptide during the simulations. Moreover, the Rg and SASA values are considered indicators of the general tertiary structure of a protein or protein-peptide/ligand complex. The Rg curve (Fig. S3c) indicates structure compactness, while the SASA curve (Fig. S3d) indicates the total exposed area (both hydrophobic and hydrophilic). The average Rg value for the M^pro^-BRIP complex for the whole simulation was 2.25 nm. The Rg graph was stable throughout the simulation, with a small increase in Rg values at around 200 ns. The Rg results showed that the structure maintained its compact conformation throughout the simulation. Similarly, we observed no major change in SASA values of the M^pro^-BRIP complex. The average SASA value for the protein-peptide complex was 55.93 nm^2^ for the whole simulation run. The structural properties indicated that the M^pro^-BRIP was stable and apt for further computational analyses.

### Role of charge distribution at the binding interface

**Figure 4:**
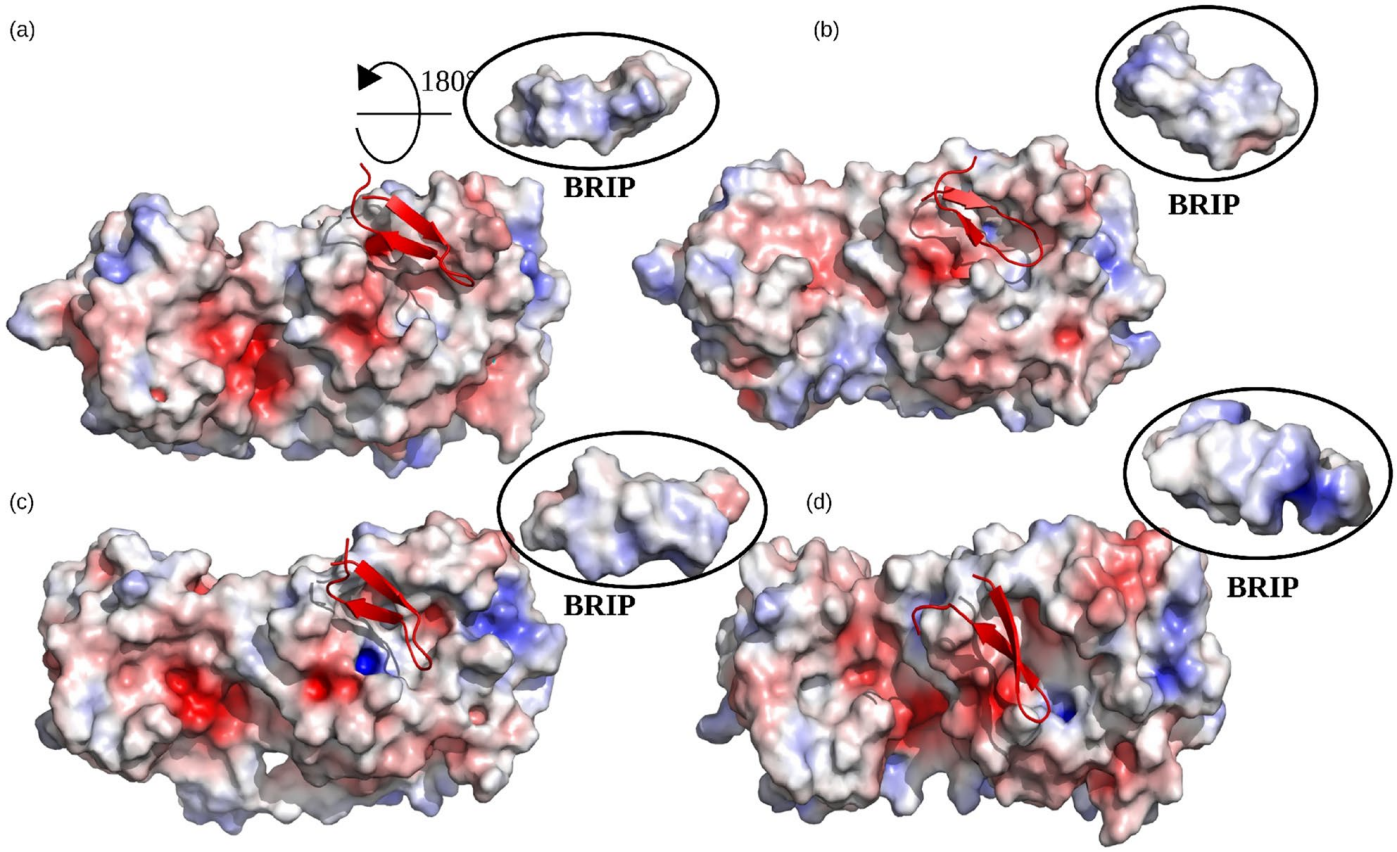
The net charge distribution on surface of M^pro^ and BRIP at different time intervals during the simulation. (**a**) 250 ns, (**b**) 500 ns, (**c**) 750 ns, and (**d**) 1000 ns.

The overall stability of protein complexes is governed by many factors, such as hydrogen bonding, packaging of hydrophobic core, and changes in secondary structures^31,32^. Moreover, the charge-charge interactions were also shown to render stability to the binding interfaces of many proteins^33,34^. We visualized the charge distribution at the binding interface of M^pro^ and BRIP peptide at different time intervals (250 ns, 500 ns, 750 ns, and 1000 ns) during the simulation (Fig. 4). The negatively charged Glu166 surrounded by neutral amino acids imparted a net negative charge to the binding interface of M^pro^. On the other hand, the positively charged Arg9 of BRIP faced the binding interface, while the negatively charged Glu6 was positioned on the outer surface. This imparted a net positive charge to the BRIP binding interface. The opposite charges present on the binding surfaces of BRIP and M^pro^ contributed to the stable binding of both the structures.

### Most stable conformation revealed by the Gibbs free energy

**Figure 5:**
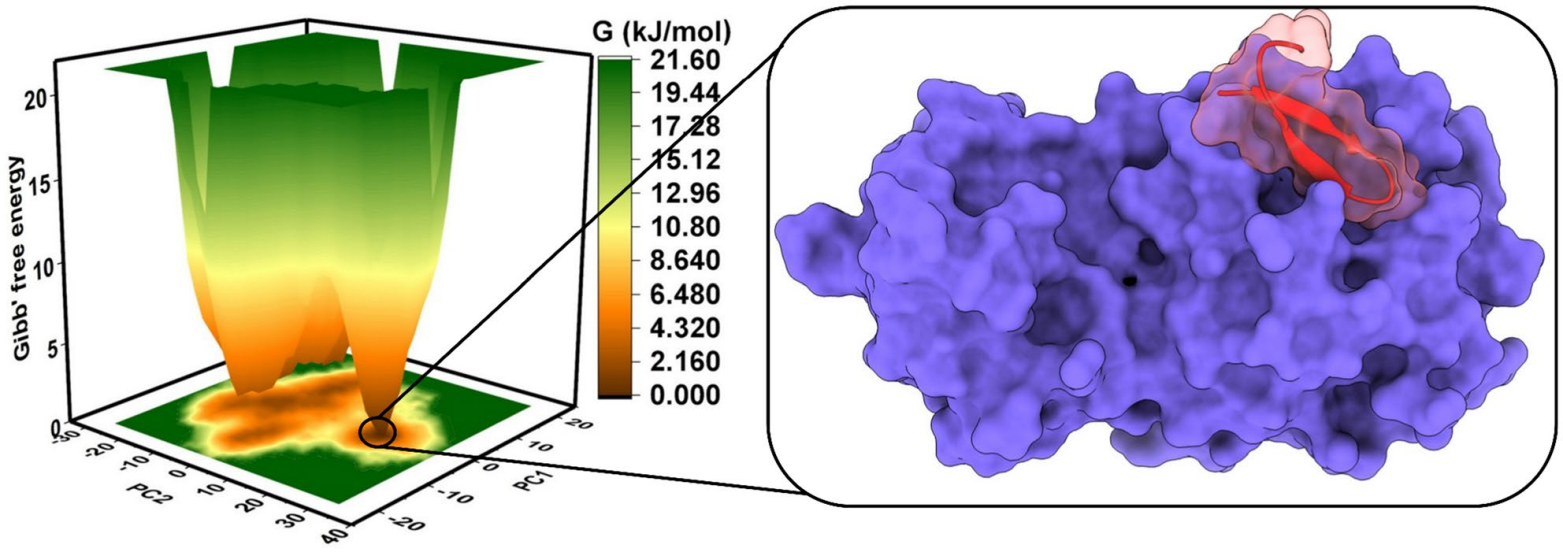
The Gibbs free energy landscape of M^pro^-BRIP complex showing the metastable conformation at the lowest energy basin.

The spatial positions of atoms of a protein structure were inspected by the analysis of the Gibbs free energy landscape (FEL)^35,36^. The FEL was plotted between the first two principal components (PC1 and PC2), where the brown, orange and yellow represented the metastable conformations with low-energy conformations, while green signified high-energy conformations of protein-peptide structure (Fig. 5). We observed a single broad, deep basin representing the most stable conformation for the M^pro^-BRIP complex. The complex corresponding to the metastable conformation with the least energy minima was extracted from the MD data, as shown in Fig. 5. The BRIP peptide was completely covering the catalytic pocket of M^pro^ at the metastable conformation. The binding of M^pro^ and BRIP was further evaluated by calculating the post-processing thermodynamic binding free energy by a widely acceptable Molecular Mechanics Poisson-Boltzmann Surface Area (MM-PBSA) approach.

### Analysis of post-processing thermodynamic binding free energy

The MM-PBSA approach for free energy calculations is widely acceptable for predicting the binding affinity between receptors and ligands/peptides^37,38^. It utilizes the polar and apolar solvation parameters to derive the final binding free energy^37^. The components of the final binding free energy are Van der Waals, electrostatic, polar solvation, and SASA energies. Except for the polar solvation energy, all other components contributed favorably to the protein-peptide binding. The most significant contributions were made by Van der Waals (− 176.495 kJ/mol) and electrostatic (− 94.801 kJ/mol) energies. The total binding free energy of the system was − 136.106 kJ/mol. The final binding free energy for the entire simulation period is shown in Fig. S4. The low binding free energy supported by *in-vitro* results thereby warrants the development of BRIP as a potential inhibitor of the M^pro^ of SARS-CoV-2.

### Detecting the key residues involved in M^pro^-BRIP binding

**Figure 6:**
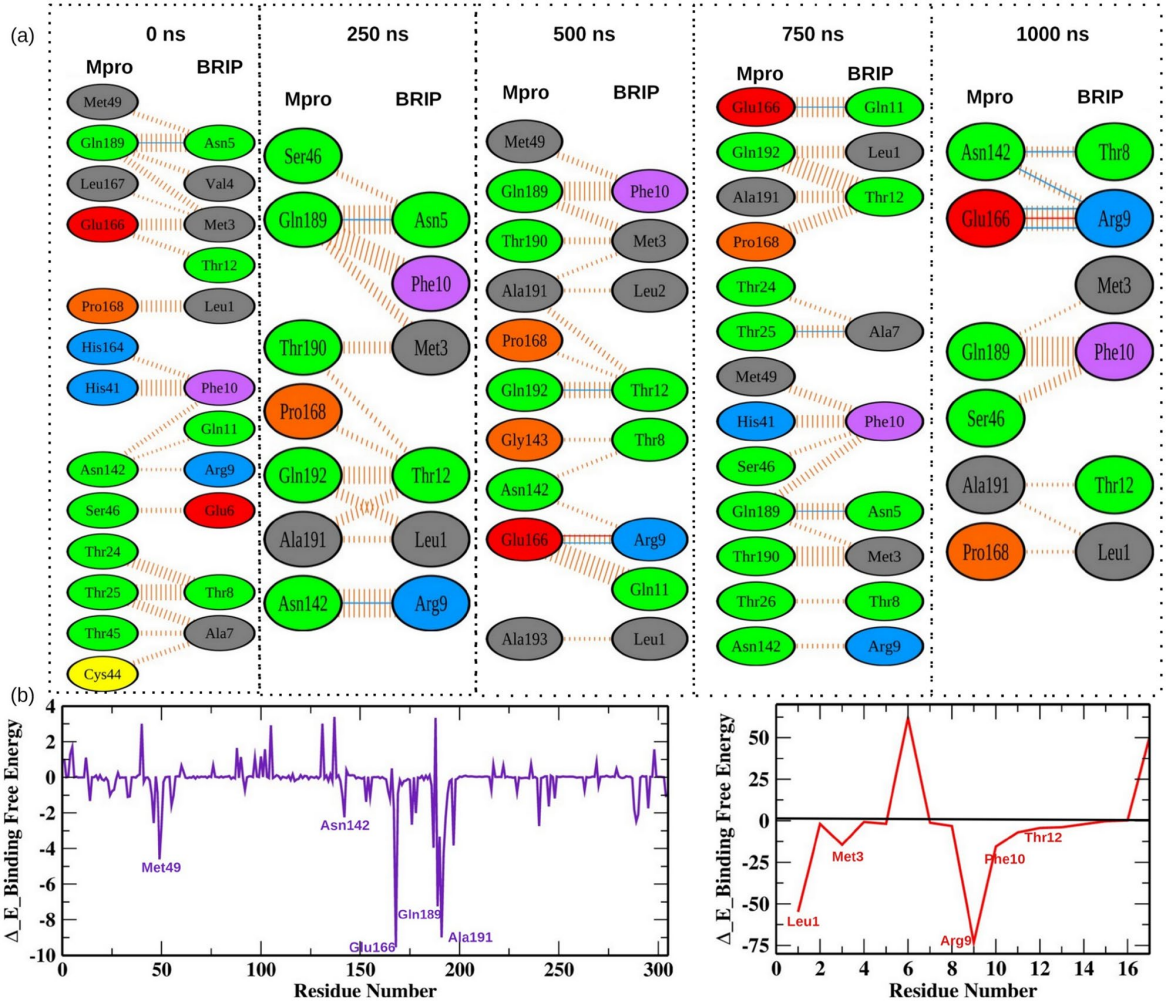
The key residues involved in M^pro^-BRIP interactions. (**a**) The interaction profiles of static poses extracted at different time intervals during the simulation. The solid blue and red color lines represents hydrogen bonds and salt bridges respectively, while the striped lines denote non bonded interactions. The residue color coding scheme is as follows: positive (blue), negative (red), neutral (green), aliphatic (gray), aromatic (purple), proline and glycine (orange). (**b**) The per residue contribution energy of M^pro^ and BRIP observed for the entire simulation run by the MM-PBSA method.

We extracted several snapshots of M^pro^-BRIP complex at time intervals (0, 250, 500, 750, and 1000 ns) from MD trajectories. We analyzed the molecular interactions between both the M^pro^ and BRIP, as shown in Fig. 6a. The BRIP peptide formed a hydrogen bond with residue Gln189 for the first 250 ns of the simulation, thereafter; Glu166 was involved in hydrogen bonding with BRIP till the end of the simulation. The residue Gln189 also showed a hydrogen bond at 750 ns of the simulation run. Apart from it, the residues Asn142, Gln192, and Thr25 also formed hydrogen bonds at different time intervals during the simulation. The residue Glu166 also interacted with BRIP by showing salt bridge interactions at 500 ns and 1000 ns. All these residues were shown to interact with small molecules developed to inhibit the M^pro^ of SARS-CoV-2^39–41^. To further strengthen our observations, we decomposed the total binding free energy into per residue contribution energy, as shown in Fig. 6b. The contribution energy results showed that the residues involved in hydrogen bonds, salt bridge formations, and non-bonded contacts showed lower binding free energies, thereby validating the critical role of these residues in M^pro^-BRIP interactions. Several mutations have been reported in M^pro^ protein of different lineages of SARS-CoV2. Therefore, we further wanted to understand if these mutations impact M^pro^-BRIP interaction. We focused on the mutations reported in current circulating lineages of SARS-CoV-2, i.e., Delta and Omicron. The mutations reported in M^pro^ protein of the Delta variant are at positions 1, 4, 7, 12, 14, 122, 125, 166, 285, 286, and 298^42^. Among these, mutation of Glu166 could only have an impact on M^pro^-BRIP binding. The prevalent mutations found in M^pro^ of the Omicron variant are at positions 135, 842, and 856 (retrieved from nextstrain; https://github.com/nextstrain/ncov). Recently a mutation P132H is also reported in M^pro^ protein of the Omicron variant^43^. None of these residues interacted with the BRIP peptide, suggesting it to be a promising agent.

## Conclusion

World over, researchers are exploring therapeutic molecules and peptides against SARS-CoV-2. Here, we have elucidated the potential therapeutic role of the known plant toxin family, RIPs against it. The short segment of 17AA encoding ricin domain from RIP1 of *Hordeum vulgare* (BRIP) showed potential inhibitory activity towards M^pro^ protein of SARS-CoV-2. A limited number of protein-peptide structures have been experimentally solved compared to the protein–ligand complexes for the anti-COVID drug finding. The computational analysis revealed that the Van der Waals and electrostatic energies were the major contributors to the protein-peptide (M^pro^–BRIP) interactions. *In-silico* investigations shed light on the potential mechanism of action of BRIP against M^pro^. As the peptide was retrieved from barley seed proteins, it should be safe, and the cytotoxicity studies confirmed no cytotoxic effect of BRIP at a concentration that could inhibit M^pro^. This study opens the gateway of possibilities to find anti-COVID solutions in nature-inspired therapeutic peptides.

## Methods

### RIP sequences alignment and peptide designing

The known antiviral ribosomal inactivating proteins PAP-S1 from *Phytolacca americana* (accession number KT630652.1), saporin from *Saponaria officinalis* (accession number CAA41948.1), trichosanthin from *Trichosanthes kirilowii* (accession number AAA34207.1), and an antifungal RIP1/PSI II from *Hordeum vulgare* (accession number KAE8814914.1) were retrieved from NCBI database and aligned using CLUSTAL Omega as an alignment tool. The RIP protein sequences were scanned for the conserved ricin/shiga toxin domain using the ExPASy PROSITE server (https://prosite.expasy.org/). The ricin/shiga domain-based peptides PAP from *Phytolacca americana*, SAP from *Saponaria officinalis,* TRI from *Trichosanthes kirilowii,* and BRIP from *Hordeum vulgare* were analyzed in the present study.

### Physicochemical properties analysis of peptides

The physicochemical properties of the peptides like grand average of hydropathicity (GRAVY), aliphatic index, instability index, estimated half-life, extinction coefficients, theoretical pI, and molecular weight were predicted using the ExPASy ProtParam tool^44^. The allergenicity of peptides was predicted using an online web tool- AlgPred (http://crdd.osdd.net/raghava/algpred/). The toxicity of the peptides was also predicted using the ToxinPred server^45^. A peptide with the lowest allergenicity, BRIP "LLMVNEATRFQTVSGFV" was synthesized from Helix Biosciences, India, and used for further characterization.

### In vitro inhibition assay of M^pro^

MBP-tagged (SARS-CoV-2) assay kit was used (Cat. No. Catalog #79955-1; BPS Bioscience, USA) to study the inhibition of M^pro^ by BRIP. In brief, the reactions were performed in a final assay volume of 50 µl in a 96 well plate wherein each reaction consisted of 30 µl of M^pro^ (150 ng) prepared in an assay buffer containing 1 mM DTT, 10 μl of fluorogenic protease substrate (DABCYL-KTSAVLQSGFRKME-EDANS) to a final concentration of 50 μM. BRIP (0.25–50 nM) was added as an inhibitor of M^pro^. Appropriate assay control by replacing inhibitor with inhibitor buffer was included. The plate was sealed with the plate sealer and incubated overnight at RT. The fluorescence intensity was measured in a microtiter plate-reading fluorimeter (Biotek Synergy™ H1, USA) with an excitation wavelength of 360 nm and detection of emission at a wavelength of 460 nm. All the values were corrected by subtracting the blank values. The percent inhibition was calculated using the following formula:

$$\% \;{\text{inhibition}} = \left[ {\left( {{\text{assay}}\;{\text{control}} - {\text{test}}\;{\text{inhibitor}}} \right)/{\text{assay}}\;{\text{control}}} \right] \times {1}00$$

To calculate the inhibitory concentration (IC_50_) value, BRIP was tested at concentrations of 0.25, 0.5, 1, 5, 10, 25, 50 nM against M^pro^ protein. The inhibitor concentration was plotted against the percent inhibition, and the IC_50_ value was calculated using the non-linear regression equation of the resulting graph using GraphPad Prism software version 8.0.2.

### Hemolytic activity of the peptide

The rat erythrocytes were used for conducting the cytotoxicity studies. All experimental protocols were approved by Institutional Animal Ethics Committee of CSIR-Institute of Himalayan Bioresource Technology (IAEC, CSIR-IHBT; IAEC/IHBTP-4/Mar 2021). The experiments were performed in accordance with relevant guidelines and regulations. In addition, the experiments were in compliance to the ARRIVE guidelines. The RBC preparation and hemolysis studies were performed following Deller et al.^46^. Briefly, the blood from rats (1 ml) was centrifuged at 1950×*g* for 5 min at room temperature (24 ± 2 °C). The plasma was removed by removing the top layer and was replaced with an equal volume of PBS. For toxicity studies, RBCs with BRIP (1 µg/ml, 10 µg/ml, 50 µg/ml, and 100 µg/ml) in PBS were evaluated for hemolysis studies. The RBC samples with 0% and 100% hemolysis were prepared by adding PBS (500 µl) to RBC (500 µl) suspension and H_2_O (500 µl) to RBC (500 µl) suspension, respectively. The desired RBC suspension (40 µl) was added to 400 µl PBS and centrifuged at 1,000 g for 5 min at 4 °C. The supernatant (50 µl) was diluted in 150 µl of PBS, and absorbance was measured at 450 nm. Hemolysis percent was calculated using the following equation:

$${\text{Hemolysis }}\% \, = \frac{{{\text{Abs}}_{{{\text{sample}}}} - {\text{Abs}}_{{0\% {\text{hemolysis}}}} }}{{{\text{Abs}}_{{{1}00\% {\text{hemolysis}}}} }} \times 100$$

The concentrations at which hemolysis was observed to be less than 10% were considered as non-toxic, while those showing 10% to 49% were considered slightly toxic, as previously reported^47,48^.

### Structure prediction of BRIP

The 3D structure of BRIP was modeled using an online homology-modeling tool: I-TASSER (https://zhanggroup.org/I-TASSER/ ), which models the target protein based on templates from RCSB-PDB using multiple threading alignments^49^. The models were retrieved based on C-score, estimated TM-score, and RMSD. Further, the models were refined using Galaxy refine server (http://galaxy.seoklab.org/cgi-bin/submit.cgi?type=REFINE). Finally, the reliability of the models was checked by Ramachandran plot analysis using an online web server by MolProbity^50^. The most accurate model was selected for further study.

### Binding site identification and molecular docking

The experimentally resolved co-crystal structure of the M^pro^ with 2.16 Å resolution (PDB ID: 6LU7)^4^ was scanned for the identification of potential binding sites by the Discovery studio package^23^. A total of five potential binding sites were identified. The BRIP peptide was docked on all the five potential binding sites by following the zdock procedure in the discovery studio. The M^pro^ structural coordinates were fixed, while the BRIP peptide was allowed to move around the potential binding sites. The docking parameters were kept at default, as defined in our previous study^51^. For visualization of docking results, the DIMPLOT program of the LigPlot + suite was utilized^52^.

### Steered MD simulations

The GROMACS package (version 2018.1)^53,54^ was used for SMD simulations to identify the binding pose with the highest binding affinity. SMD is a promising tool for making comparisons between the rupture force and affinity of a ligand for its target protein^55^. All the five binding poses were subjected to SMD simulations by placing the protein-peptide complex at coordinates (4.2, 4.1, 3.0) within a simulation setup consisting of a rectangular box. The size of the simulation box was 8.5*8.3*25 Å. The protein-peptide topologies were prepared by GROMOS96 43a1^56^ force field by in-built scripts of GROMACS software. The simulation box was filled with a simple point charge (SPC) water model. An appropriate number of Na^+^ and Cl^-^ ions were added to neutralize the system. The steepest descent algorithm was used for energy minimization. Afterward, the peptides were pulled out of each binding site by applying a spring constant of 250 kJ/mol/nm^2^ at a constant velocity of 0.01 nm/ps.

### Conventional MD simulations and binding free energy calculations

The binding pose of M^pro^-BRIP peptide with the highest affinity was subjected to conventional MD simulations (1 μs) by utilizing the GROMACS package. The GROMOS96 43a1^56^ force field was applied for obtaining M^pro^ topology. The protein-peptide complex was solvated in a cubic box with periodic boundary condition by a simple point charge water model. Na^+^ and Cl^-^ ions were added to the simulation box to neutralize the system. The initial steric clashes were removed by subjecting the protein-peptide complex to energy minimization (steepest descent method) for 1000 steps at a tolerance cut-off of 10 kJ/mol/nm. The equilibration was achieved in two steps (NVT and NPT), each executed for 1000 ps. The reference temperature for simulations was set at 300 K by the modified Berendsen thermostat (V-rescale), while 1 bar of reference pressure was maintained during the simulation through the Parrinello-Rahman pressure-coupling method. The length constraints were defined by the LINCS algorithm for covalent bonds, while the long-range electrostatic interactions were computed by the particle-mesh Ewald method. We used the leap-frog md integrator, 1 nm cut-off values for the vdW and Coulomb energy, and the values were recorded after every 10 ps. The rest of the MD parameters used in the study were explained comprehensively in our previous studies^51,57,58^. The root mean square deviations (RMSD) and free energy landscape (FEL) were calculated by exploiting the in-built algorithms of the GROMACS package. The FEL was plotted by origin software, while all other graphs 2D graphs were drawn by the GRACE toolkit. The post-processing, end-state thermodynamic binding free energy of the M^pro^-BRIP structure was calculated by the Molecular Mechanics Poisson-Boltzmann Surface Area (MM-PBSA) methodology^37^, which utilizes the following equation:

$$\Delta {\text{G}}_{{{\text{binding}}}} = {\text{G}}_{{{\text{complex}}}} - \left( {{\text{G}}_{{{\text{receptor}}}} + {\text{G}}_{{{\text{peptide}}}} } \right)$$

where ΔG_binding_, G_receptor_, and G_peptide_ depict the total free energy of the complex, receptor, and unbound peptide.

The equation stated above is valid for the protein–ligand and protein-nucleic acid complexes.

## Supplementary Information

Supplementary Information 1.

Supplementary Video 1.

Supplementary Video 2.

Supplementary Video 3.

Supplementary Video 4.

Supplementary Video 5.

## Data Availability

The datasets analyzed during the current study are available in the NCBI database (https://www.ncbi.nlm.nih.gov/), accession numbers KT630652.1, CAA41948.1, AAA34207.1 and KAE8814914.1. All the other relevant data are included in the article and its supplementary information files.
