# Supplementary material for: A ricin-based peptide BRIP from Hordeum vulgare inhibits Mpro of SARS-CoV-2
Source: Sci Rep. 2022 Jul 27;12:12802. doi: 10.1038/s41598-022-15977-y (PMC9326418; doi:10.1038/s41598-022-15977-y)

Supplementary information

Journal name: Scientific Reports

Research article: A ricin-based peptide BRIP from *Hordeum vulgare* inhibits M^{pro} of SARS-CoV-2

Author names

Prakriti Kashyap[1#], Vijay Kumar Bhardwaj[1,2,3#], Mahima Chauhan[1,2], Varun Chauhan[4], Asheesh Kumar[1,2], Rituraj Purohit[1,2,3**], Arun Kumar[1,2*], Sanjay Kumar[1**]

Affiliations:

[1]Biotechnology Division, CSIR-Institute of Himalayan Bioresource Technology, Palampur, Himachal Pradesh, 176061, India

[2]Academy of Scientific and Innovative Research, Ghaziabad, 201002, Uttar Pradesh, India

[3]Structural Bioinformatics Lab, CSIR-Institute of Himalayan Bioresource Technology, Palampur, Himachal Pradesh, 176061, India

[4]Covid-19 Testing Facility, Dietetics & Nutrition Technology Division, Council of Scientific and Industrial Research-Institute of Himalayan Bioresource Technology (CSIR-IHBT), Palampur (H.P), India, 176061

[#]Equal contribution

Corresponding author*:

Dr. Arun Kumar: arunkumar@ihbt.res.in

Co-corresponding authors**:

Dr. Rituraj Purohit: rituraj@ihbt.res.in

Dr. Sanjay Kumar: sanjaykumar@ihbt.res.in

Supplementary Table S1: Physicochemical properties of peptides.

Peptide	Molecular weight	Theoretical pI	Formula	Extinction coefficients (M-1 cm-1, at 280 nm measured in water)	Estimated half-life (mammalian reticulocytes, in vitro)	Instability index	Aliphatic index	Grand average of hydropathicity (GRAVY)	Allergenicity scores (Threshold= -0.4)
**PAP**	2026.34	6.14	$C_{90}H_{144}N_{24}O_{27}S_1$	0.735	20 hours	28.88	91.76	-0.135	0.17
**SAP**	2052.42	8.75	$C_{92}H_{150}N_{26}O_{25}S_1$	0.726	20 hours	3.28	114.71	0.265	0.037
**TRI**	2012.25	6.14	$C_{89}H_{142}N_{24}O_{29}$	0.740	20 hours	90.66	80.59	-0.565	-0.17
**BRIP**	1912.23	6.00	$C_{86}H_{138}N_{22}O_{25}S_1$	-	5.5 hours	9.41	102.94	0.700	-0.57

Supplementary Table S2: The interacting residues of M^{pro} protein belonging to five predicted binding sites with BRIP peptide.

M^{pro}-BRIP Complex	H-bonds	Non bonded contacts
**Binding Site 1**	His41 and Gln19	Thr29, Thr26, Met49, Ser46, Thr26, Asn119, Asn142, Cys146, Gln169, Gln69, and Thr21
**Binding Site 2**	Gln189	His164, Glu166, Met165, His41, Asn142, Cys44, Thr45, Ser46, Pro168, Met49, Thr24, and Thr25
**Binding Site 3**	Thr25	Gly143, Asn142, Phe140, Leu141, His172, Ser139, Gly138, Glu166, Cys145, Thr45, Ser46, Thr26, and Leu167
**Binding Site 4**	Tyr237 and Ala285	Tyr239, Thr199, Leu272, Asn238, Asn236, Met276, Leu286, and Gly278
**Binding Site 5**	Asp245	Ile249, Gln244, Thr243, Gln107, Gln110, and Asp248

Supplementary Fig. S1: Ramachandran plot results of refined BRIP model.

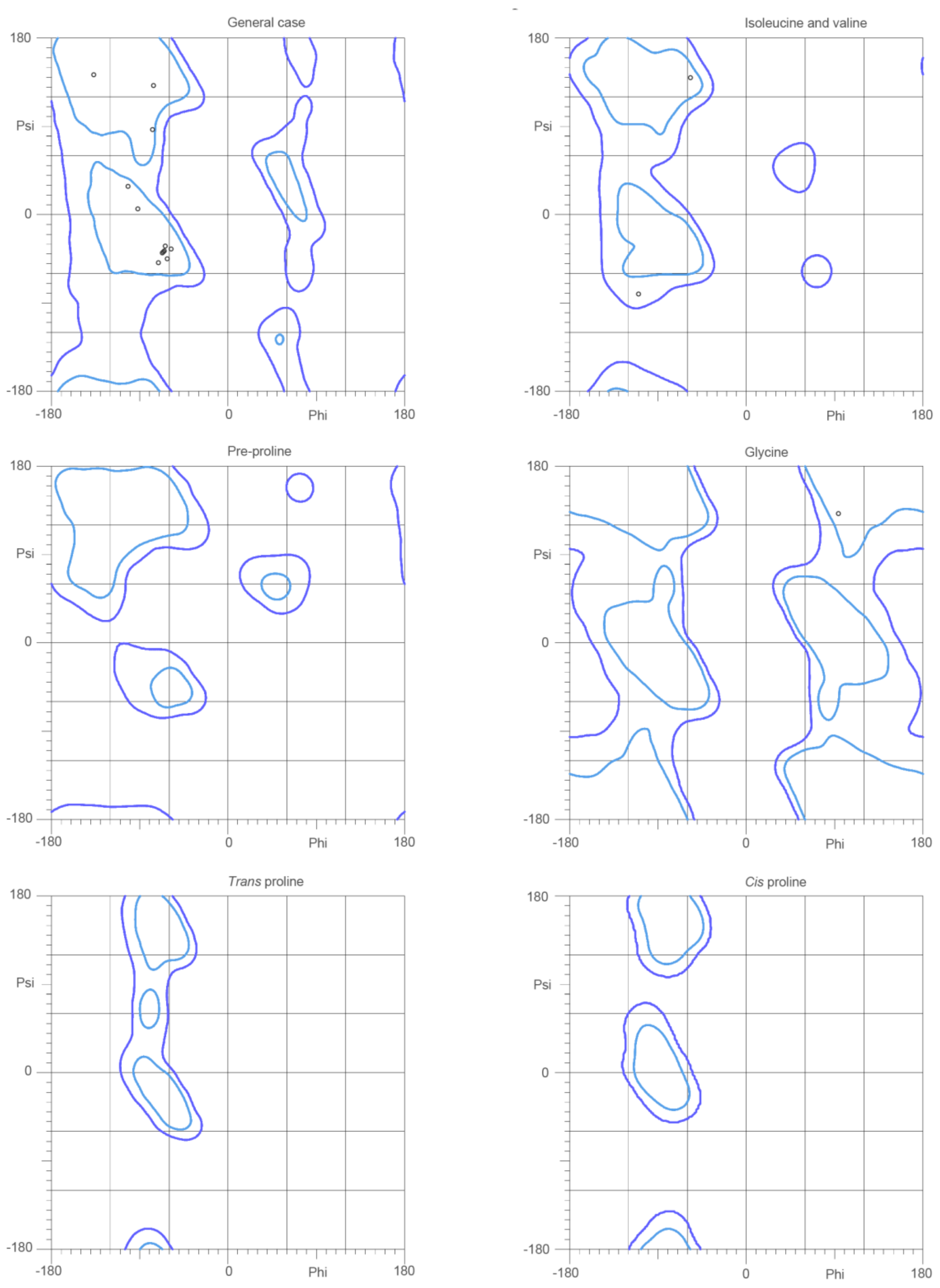

93.3% (14/15) of residues were in favoured (98%) region.
100.0% (15/15) of all residues were in allowed (>99.8%) regions.
There were no outliers.

Supplementary Fig. S2: The relationship between pull force and distance moved by the BRIP peptide during steered MD simulations.

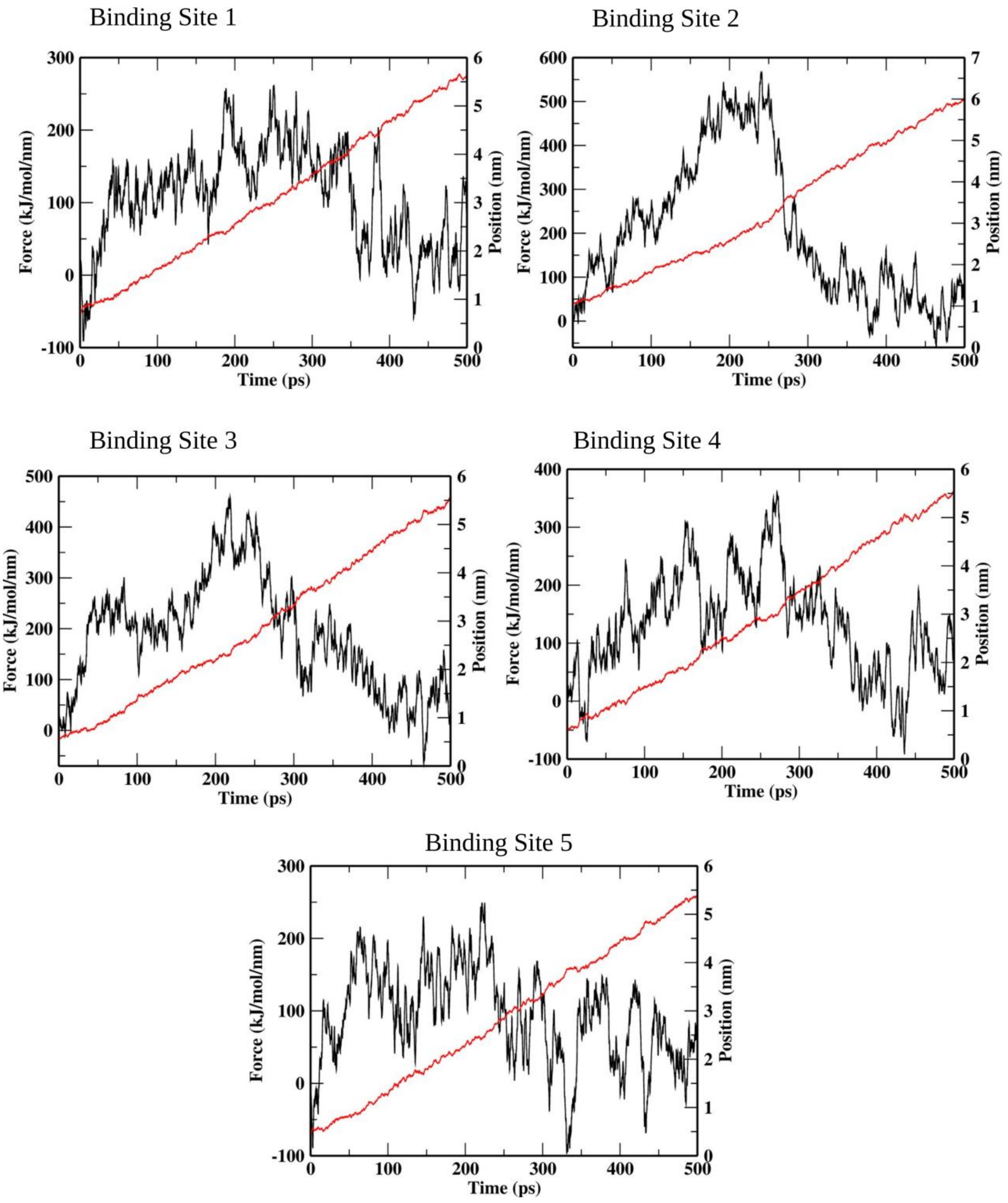

Supplementary Fig. S3: Analysis of structural parameters of M^{pro}-BRIP complex. (a) RMSDs for M^{pro}-BRIP complex. The green line represents the running average. (b) RMSF of backbone C-α atoms (c) Rg of M^{pro}-BRIP complex and (d) SASA of M^{pro}-BRIP complex.

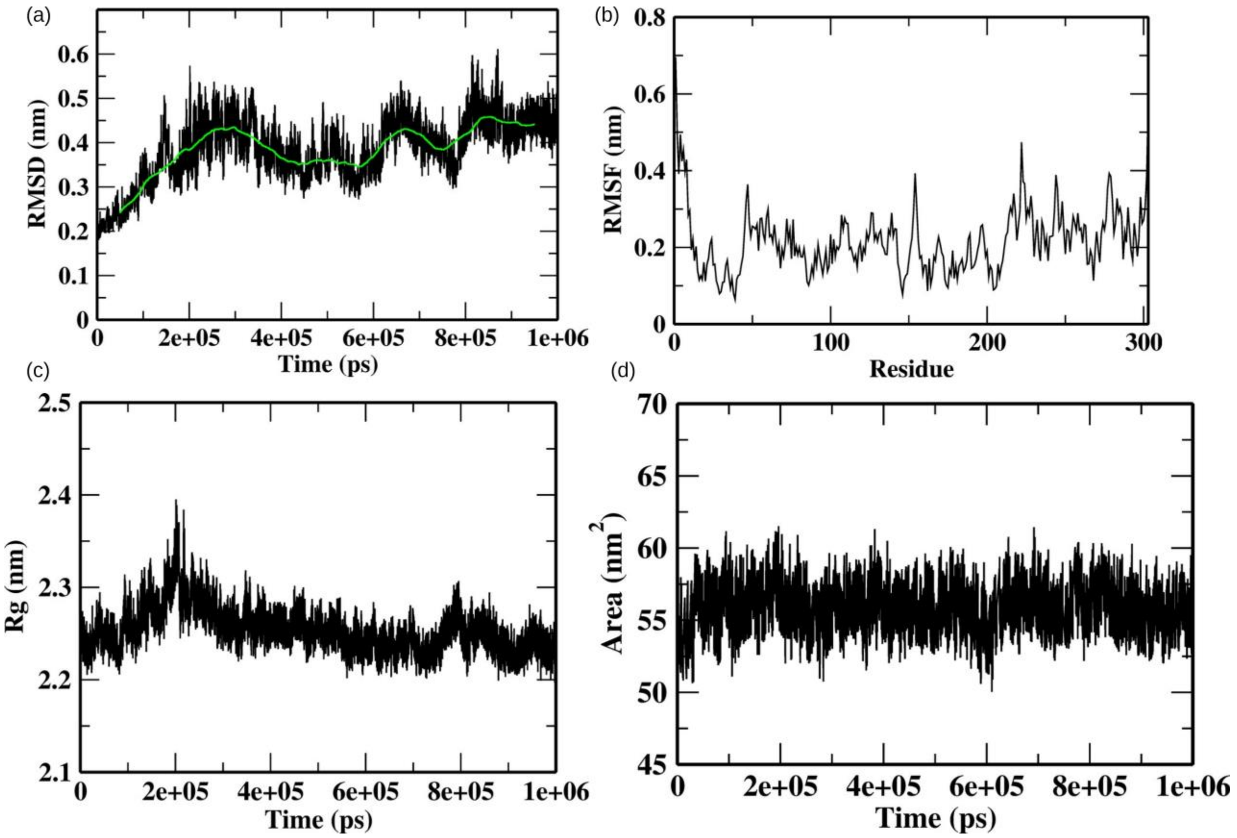

Supplementary Fig. S4: Graphical representation of the Delta_E_Binding free energy for M^{pro}-BRIP complex.

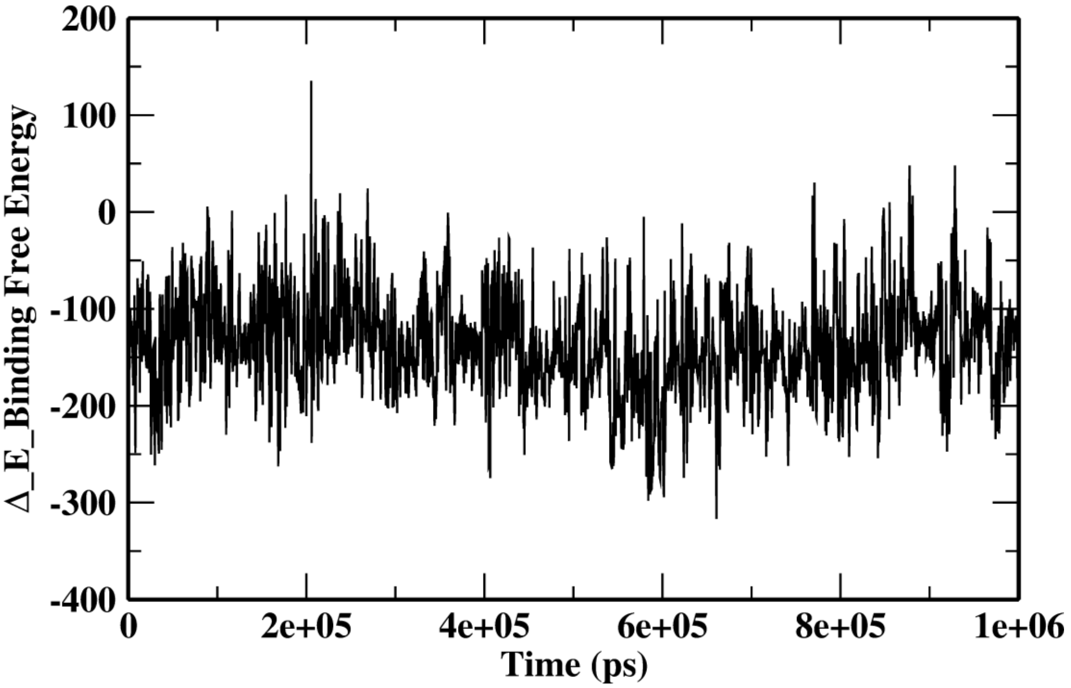

Supplement: Supplementary file 1 — Supplementary Information 1. [file 41598_2022_15977_MOESM1_ESM.pdf]
